# Ionization efficiency prediction of electrospray ionization mass spectrometry analytes based on molecular fingerprints and cumulative neutral losses

**DOI:** 10.1186/s13321-025-01129-7

**Published:** 2025-12-06

**Authors:** Alexandros Nikolopoulos, Denice van Herwerden, Viktoriia Turkina, Anneli Kruve, Melissa Baerenfaenger, Saer Samanipour

**Affiliations:** 1 Van ’t Hoff Institute for Molecular Sciences (HIMS), University of Amsterdam, 1098XH Amsterdam, The Netherlands; 2 Department of Materials and Environmental Chemistry, Stockholm University, 11418 Stockholm, Sweden; 3 Division of BioAnalytical Chemistry AIMMS Amsterdam Institute of Molecular and Life Sciences, Vrije Universiteit Amsterdam, 1081 HZ Amsterdam, The Netherlands; 4 UvA Data Science Center, University of Amsterdam, 1012 WP Amsterdam, The Netherlands; 5 Queensland Alliance for Environmental Health Sciences (QAEHS), The University of Queensland, Brisbane, QLD 4072 Australia

**Keywords:** Semi-quantification, Electrospray ionization, Mass spectrometry, Environmental analysis

## Abstract

Quantification is a challenge for non-targeted analysis (NTA) with liquid chromatography–high resolution mass spectrometry (LC–HRMS), due to the lack of analytical standards. Quantification via structure-based predicted ionization efficiency (IE) has been shown to provide the highest accuracy in estimating concentration. However, achieving confident analyte identification is a challenging task, as multiple candidate structures may be likely. This uncertainty in identification limits the reliability of structure-based IE prediction models, since quantification can be severely compromised in cases of wrongly (tentatively) identified chemicals or lack of candidate structures. Here we investigate the possibility of using cumulative neutral losses from fragmentation spectra (i.e. MS2) to predict the log*IE*. The first model was based on molecular fingerprints and was applied on structurally identified analytes. PubChem fingerprints performed the best with the root-mean-square error (RMSE) of 0.72 log*IE* for the test set. The second model was based on the MS2 spectrum, expressed as cumulative neutral losses. This approach is applicable to analytes with unknown structures and showed promising results with RMSE of 0.79 log*IE* for the test set and 0.62 log*IE* for chromatographic features extracted from LC-HRMS data of tea extracts spiked with pesticides. The prediction models were compiled in a Julia package, which is publicly available on GitHub, and may be used as part of a quantification workflow to estimate concentrations of identified and unidentified compounds in NTA.

**Scientific contribution:** This study expands the possibilities of standard free quantification for HRMS. It aims to provide reliable IE prediction for known substances by robust fingerprint calculation, and more importantly IE prediction for unknown substances using their MS2 fragmentation pattern. These workflows employ minimal method-specific variables, highlighting the tool generalizability.

**Supplementary Information:**

The online version contains supplementary material available at 10.1186/s13321-025-01129-7.

## Introduction

Non-targeted analysis (NTA) has revolutionized the field of analytical chemistry by employing the exceptional resolving power and sensitivity of liquid chromatography–high resolution mass spectrometry (LC–HRMS) [1–3]. NTA aims to acquire a profile covering a broad range of compounds and to provide a comprehensive sample overview. Due to its capabilities, NTA has been applied in many fields, such as exposomics [4], metabolomics [5] and biomarker discovery [6], in which complex samples need to be analysed in a non-targeted approach.

Targeted analysis is a more conventional approach for accurate quantification, since analytical standards are used to create compound-specific calibration curves. The need for compound-specific calibration curves originates from the non-universality of the response factor (i.e. the signal to concentration ratio), which is a known issue since the introduction of electrospray ionization mass spectrometry (ESI-MS) [7, 8]. The differences in analyte responsiveness are associated with the ionization process in the ESI source [9].

While quantification is a relatively easy task for targeted analysis and provides accurate results when analytical standards are available, it remains one of the major challenges for NTA. The correlation of ionization efficiency (IE), expressed as the efficiency of generating gas-phase ions from analyte molecules or ions in the ESI source [10], with the response factor provides a quantitative approach to estimate analyte concentrations in NTA. The factors which affect IE have been investigated extensively in previous studies [11–15] and can be summarized in four categories: (1) compound structure, (2) eluent composition, (3) instrumental setup, and (4) matrix effects [16, 17].

Previous studies have mainly focused on the implementation of molecular descriptors for the development of quantitative structure-property relationship (QSPR) models to predict IE [8, 18, 19]. Molecular descriptors describe a physicochemical or structural property of a molecule, such as the total number of atoms (2D), molecular weight (2D), XlogP (2D), and van der Waals volume (3D). Several properties are calculated based on the molecule at a low-energy geometry, which is estimated in structure optimization [20]. Nevertheless, it is uncertain whether the lowest possible energy configuration has been reached for the descriptors calculation, especially for molecules of large size and complexity. This may result in potentially unreliable data [21–24].On the other hand, molecular fingerprints (FPs), deterministically, encode molecular structures in vectors, which contain specific structural information. Additionally, FPs are generally more interpretable than descriptors, resulting in more transparent models while maintaining comparable performance to descriptor-based models [24–27].

Apart from structural information, most IE prediction models in literature require additional method-specific variables, such as descriptors for the mobile phase [27, 28]. Alternatively, the models are trained on datasets based on one analytical method [26, 29]. However, the required metadata might not be available in some cases (e.g. retrospective analysis of archived data) and therefore such approaches lead to limited model applicability.

Despite persistent efforts and advancements in data processing tools, identification is still the primary challenge of NTA with LC-HRMS [30–36]. Even with MS2 spectral information, the library matches often result in multiple possible candidates for each feature and therefore uncertain annotations due to limited available spectra and structural information present in those spectra. Predicting IE based on structural information in such cases would add another level of uncertainty to the predicted value.

A recent study [27] demonstrated the potential of indirectly using mass spectral data by predicting a probabilistic molecular fingerprint employing SIRIUS+CSI:FingerID, which were used for prediction of *IE* values. The prediction accuracy of such models is highly dependent on the accuracy of the molecular formula calculation and molecular fingerprint prediction. On the other hand, *IE* could be directly predicted from MS2 spectral fragments. The fragments can be expressed in the form of cumulative neutral losses (CNLs), which are the mass differences between the precursor ion and each fragment. Previous studies [21, 37, 38] have shown that CNLs create low-dimensional data and retain the structural information of an MS2 spectrum, thus supporting a more effective capture of important structural patterns.

For this purpose, the current study aimed to develop a workflow for IE prediction. The first part, which addresses compounds with a known structure, predicts log*IE* based on structural information, expressed in the form of a molecular fingerprint, and sets up a baseline for IE predictions. The second part, which addresses unidentified analytes, predicts log*IE* based on fragments from MS2 spectra directly, which are expressed as CNLs. As it is considered essential for IE prediction [13, 14], the pH of the aqueous phase of the eluent was incorporated as an additional variable to the models. Each workflow part consists of a gradient-boosted trees model providing the predicted log*IE* value.

## Methods

### Overall workflow

**Fig. 1:**
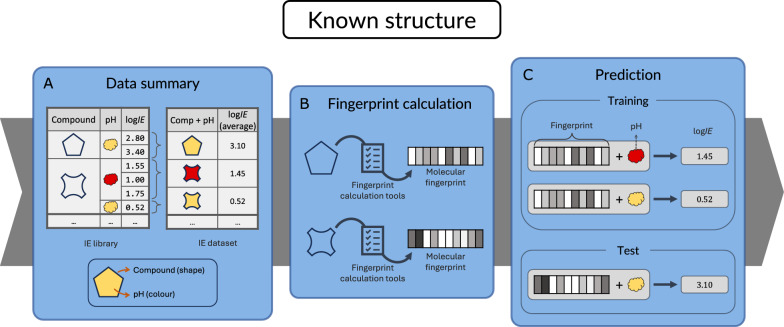
Graphical workflow for structure-based IE prediction. The fingerprint approach is based on molecular fingerprints and addresses molecules with known structures. The log*IE* values are averaged by compound and pH value (**A**). Structures are converted to fingerprints (**B**), which, in combination with the pH, are used for the model training and testing (**C**)

In this study, a general workflow was designed to predict *IE*. The workflow consists of two independent models, which predict log*IE* in different ways. The first approach (Fig. 1) was designed for compounds with a known molecular structure, for which a structure-based model was developed. Sixteen sets of molecular fingerprints, e.g. Chemistry Development Kit (CDK) and Molecular Access System (MACCS), were calculated using the canonical SMILES from the structures of unique compounds with known *IE*s. A model was trained on the fingerprints and the pH of the aqueous phase of the eluent to predict log*IE*. Excluded from training, 20$\%$ of the total number of compounds were used as a test set to evaluate the model performance.

**Fig. 2:**
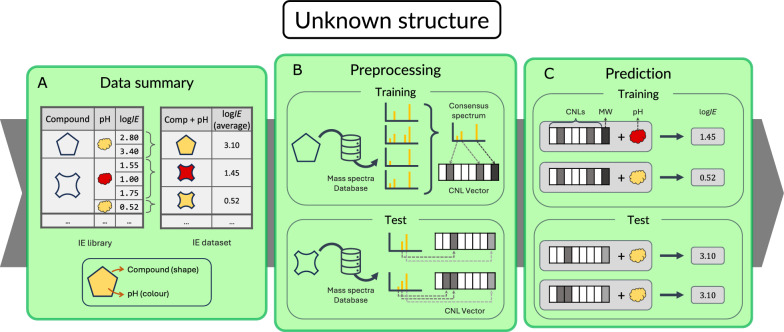
Graphical workflow for the MS2 spectrum-based IE prediction. The CNL approach is based on mass spectral information and addresses molecules with unknown structures. The log*IE* values are averaged by compound and pH value (**A**). MS2 spectra are retrieved for each compound, filtered with their consensus spectra (only the training set), and converted to CNLs (**B**). The CNLs, MW and pH are used for the model training and testing (**C**)

The second approach (Fig. 2) was designed for compounds with an unknown molecular structure, for which a MS2 based model was developed. MS2 spectra of compounds with reported *IE*s were collected from mass spectra databases and were compared to create a consensus spectrum for each compound. The consensus spectra were converted to binary vectors representing the presence or absence of common CNLs. The CNL vector, the molecular weight (MW) and the aqueous pH were used to train a model for predicting log*IE*. Excluded from training and consensus spectra filtering, 20$\%$ of the total number of compounds were used as a test set to evaluate the model performance. Each step of the workflow (preprocessing, data handling, hyperparameter optimisation, model development) is described in detail in the corresponding Methods subsections.

It should be noted that these models take either SMILES strings or MS2 spectra as input and predict log*IE* as output. For final semi-quantification these predicted *IE* need to be converted to response factors following the procedure described by Malm et al. [39].

### *IE* dataset

The first step to create the *IE* prediction models was the collection of literature data with reported *IE*s. The dataset, compiled by Sepman et al. [27], contained a collection of IEs for 1191 unique compounds measured in positive ionization mode (ESI+), which originated from the combination of thirteen previous studies and their conversion to a unified IE scale. Although the IEs are in a relative scale with methyl benzoate as the anchor compound, they will be hereinafter referred to as ionization efficiencies for simplicity. The compounds were measured in different experimental conditions, including but not limited to different mass spectrometry instruments, ESI architectures and mobile phases, resulting in a total of 6049 entries. It is important to mention that none of the entries were excluded based on their experimental parameters. For example, all reported ESI architectures, such as Agilent JetStream, Thermo Fisher Scientific HESI-II, Sciex TurboSpray, and Waters ZSpray^™^, were included in the dataset. For the modelling we only included the pH of the aqueous phase of the eluent, which has high impact on *IE* [14], and is arguably the most important variable for *IE* prediction among eluent descriptors [27, 28]. No other experimental condition was included to minimize the dependence of the developed models on experimental variables and thus to broaden their applicability.

An examination of the *IE* values of each unique compound, based on their InChI, revealed that several compounds in the dataset showed high *IE* variability. Specifically, the median *IE* range for individual compounds in the dataset was 2.0 log*IE* units, with some compounds at higher ranges (e.g. pyridine at 4.1 log*IE* units). Therefore, to provide useful model input and minimize dependence on experimental conditions, the *IE*s of each unique compound were summarized. The pH value of each measurement was rounded to the nearest whole number and measurements at the same rounded pH were grouped together. For each group a new entry was created, with a pH value equal to the average of the initial unrounded pH values, and an *IE* value equal to the average *IE* value, therefore forming a new dataset, hereinafter referred to as *IE* dataset. For every unique compound in the dataset, the SMILES and InChIKey were retrieved based on the reported InChI, using the PubChemPy package [40]. Forty three compounds were discarded, as PubChemPy was unable to retrieve their SMILES and/or InChIKey. Finally, the *IE* dataset consisted of 1148 unique compounds in a total of 1945 entries. These entries cover a wide range of pH values (1.4–12.2), with the majority of them at acidic and neutral pH (Figure S1).

A Principal Component Analysis (PCA) on the *IE* dataset was performed using the combination of six non-hashed molecular fingerprints to fully evaluate the distribution of our data. Additionally, ClassyFire [41] was employed to classify the chemicals in our dataset based on the chemical class. This further enabled us to evaluate the chemical class and the *IE* value dependency.

### CNL-*IE* dataset

The second part of the study aimed to develop an *IE* prediction model on mass spectrometry fragmentation data. For this purpose, 1.1 million mass spectra were collected from three sources: the NORMAN MassBank database [42] (accessed January 2025), the MassBank of North America (MoNA) database, and the NIST 20 library. The mass spectral data included the molecular information (molecular name, InChIKey, and exact mass) of the analyte, the instrumental metadata (resolution), and the measurement metadata (ionization mode).

The ESI+ mode spectra with at least one fragment and with resolution 5000 or better were considered to assure sufficient spectral quality. Protonated molecules, [M+H]$^{+}$, were considered due to the fact that measured *IE* values correspond to these species. Furthermore, the spectra which could not be unequivocally associated with a chemical structure due to missing InChIKeys were discarded.

The cumulative neutral losses (CNLs) of each spectrum were determined as the mass difference between the precursor ion and the fragment. While many unique CNL values are present in the spectra database, only some of them contain valuable information for *IE* prediction. Some CNL values may correspond to uncommon fragments specific for a few compounds, while others might be artifacts originating from the spectrum processing (e.g. componentization). The frequency of occurrence of each CNL in all filtered spectra was determined, resulting in 21199 unique CNLs with m/z values ranging from 1.01 to 1201.02. The CNLs were ranked based on their occurrence probability [37], and the 400 most frequent CNL values were selected keeping a balance between having a model with a sufficient number of features and removing noise. For each spectrum, a bit vector was created based on the selected CNL values, with m/z values ranging from 1.01 to 210.09. All bits which corresponded to a CNL of the spectrum with a mass tolerance of 20 mDa were set to 1, while bits which corresponded to CNLs absent from the spectrum were set to zero. Overall, the database spectra were transformed in a dataset containing molecular information and the CNL data. The CNL dataset was combined with the *IE* dataset based on InChIKeys resulting in the CNL-*IE* dataset. This dataset consisted of molecular identifiers, the log*IE* values, the CNLs, the precursor ion mass and the pH of the aqueous phase of the eluent, for 62865 spectra. Finally, the CNL-*IE* dataset was used for the CNL model training and testing. It should be noted that this strategy was necessary to overcome the issue of small and imbalanced dataset containing both experimentally defined *IE* values and MS2 spectra as well as to overcome the issues associated with data leakage.

### FP model

Twelve fingerprint types (Table 1), as well as their combination, were calculated using padelpy, a Python wrapper for PaDEL-Descriptor [43]. To include information about molecular cyclicity, the 2D atom pairs count (APC2D) fingerprints were also combined with a compressed version of the PubChem fingerprints expressing the number of molecular rings [44]. Morgan fingerprints were calculated in a 1024-bit vector with a radius of 2 using the RDKit toolkit for python [45]. Finally, an optimized set of molecular FPs based on six non-hashed fingerprints via PaDEL [46], were calculated [43, 47].

The compound list was split in a training and test subset by employing leverage stratification to ensure the compound diversity in the two subsets. Leverage is a measure of similarity between the fingerprints of a compound compared to the rest of the dataset. Low leverage values imply that the compound has high similarity to the rest of the dataset, while higher values imply that the compound is different. The leverage of each compound in the *IE* dataset was calculated by applying the selected fingerprints in Eq. 1, where $\hbox {X}_{i}$ is the variable vector for compound i, X is the variable matrix of the dataset, $\hbox {X}^{T}$ is the transpose of X and ($\hbox {X}^{T}$X)$^{-1}$ is the inverse of ($\hbox {X}^{T}$X). The distribution of leverages was created and divided in ten equal parts, for each of which a random train test split was performed at 80/20 ratio. The leverage calculation was done on the full set of the selected fingerprints, regardless of their feature importance in the built model, to provide more reliable leverage values [22]. The leverage-based stratification minimizes the impact of data distribution on the quality of the generated models. In other words, independently from sample size in each part of the population, a representative sample for both train and test sets is generated.

The regression model was trained to predict log*IE* from the fingerprints and the reported pH with CatBoost regressor [48]. CatBoost is an open-source library for gradient boosting on decision trees, a ML technique that performed well in previous studies [28, 49]. The grow policy hyperparameter was set to lossguide to develop the trees leaf by leaf. A randomized search was used for the hyperparameter optimization of the number of trees (from 100 to 1400 with an interval of 100), the minimum samples per leaf (4, 7, 10, 13, 25), the learning rate (0.05, 0.08, 0.1, 0.11, 0.15), the maximum tree depth (6, 7, 8, 9, 10), the sample rate for bagging (from 0.2 to 0.9), and the ratio of features to use at each split selection (from 0.4 to 1). A common pseudo-random number parameter, hereinafter referred to as random state, was used for reproducible results during the stratified train test split and model development and was included in the optimization process for values ranging from 1 to 5.

The best models for each fingerprint type were fitted and the accuracy scores for the test sets ($\hbox {Q}^2$) were compared. The $\hbox {Q}^2$ gives and overall representation of the error distribution without assuming a random distribution of the residuals across the model space. The model which had the highest $\hbox {Q}^2$ value was selected and used as the final FP model, for which the feature importance was calculated using the CatBoost built-in feature importance tool. Specifically, the value of each variable was iteratively changed, and the change of the prediction value was calculated on average. The features causing larger change on the predicted value are assigned higher feature importance expressed as percentages.

1 $$\begin{aligned} H_{i} = X_{i} (X^{T} X)^{-1} X_i^{T} \end{aligned}$$

### CNL model

A CNL regression model was built using the CNL data, the monoisotopic mass and the pH of the CNL-*IE* dataset (Fig. 2). The monoisotopic masses and pH values were scaled by 1000 and 14, respectively, to resolve differences in magnitude among the features. It should be noted that the scaling of features is not necessary for tree-based approaches. This was mainly done for numerical stability and the speed of calculations. The entries were filtered based on a minimum number of fragments in the spectrum, $\hbox {CNL}_{min}$. All entries with fragments fewer than $\hbox {CNL}_{min}$ were discarded.

Performing a split in training and test subsets of the dataset entries would result in data leakage. Multiple entries in the CNL-*IE* dataset correspond to the same compound, due to multiple measurements in the *IE* datasets and/or multiple spectra in the CNL dataset. Therefore, the stratified sampling was applied on unique chemicals using the leverage of the fingerprint selected in the final FP model ("FP model" section), to assure reliable accuracy evaluation as well as high chemical variability in both subsets.

To remove noise originating from improbable mass fragments, consensus spectra filtering was applied on the training subset. As suggested in a study by Luo et al. [50], the consensus spectra were created by selecting the fragments which appear in at least an adjustable percentage, $\hbox {S}_{min}$, of the parent spectra. Two different filtering algorithms were assessed during optimization. The first algorithm is based on the replacement of all spectra of a specific compound with its consensus spectrum, while the second one is based on the comparison of each spectrum with the consensus spectrum and the discard of fragments not present in the latter. The entries were filtered again based on the $\hbox {CNL}_{min}$ value and those with a low number of fragments were discarded. Despite the fact that the training subset was subjected to consensus spectra filtering, the test subset remained unchanged, since such noise filtering would not be possible in real-life samples originating from NTA experiments.

The log*IE* prediction model was trained using CatBoost regressor [48], for which the grow policy was set to lossguide. A regression model was built on the training set of the CNL-*IE* dataset, with the monoisotopic mass of the compound, the CNLs and the pH as the model input variables. The performance of the model was dependent on several preprocessing and model hyperparameters, which were optimized with a randomized search. The preprocessing hyperparameters to be optimized were the minimum number of CNLs, $\hbox {CNL}_{min}$ (ranging from 0 to 5), the consensus threshold $\hbox {S}_{min}$ (set at 0, 0.1, 0.2 and 0.25, expressed as the minimum acceptable ratio of spectra containing a specific fragment to the total number of spectra), and the consensus algorithm (either the replacement algorithm or the filtering algorithm). The model hyperparameters were the number of trees (600, 800, 1000), the minimum samples per leaf (4, 7, 10, 13, and 25), the learning rate (0.01, 0.03, 0.05, 0.07, 0.1), the maximum tree depth (6, 7, 8, 9, 10), the sample rate for bagging (ranging from 0.05 to 1.0), the ratio of features used at each split selection (ranging from 0.05 to 1.0), and the random state (ranging from 1 to 5). Every pseudo-random function in the CNL workflow used the same random seed as the random state specified in the optimization results. A log*IE* prediction model was trained with the optimal hyperparameters.

Similarly to the FP model, the CNL model accuracy scores for the test sets ($\hbox {Q}^2$) and the feature importance were calculated. The variables with the highest importance that represented CNLs were investigated and their molecular formulas were suggested. The mass accuracy was set to 20 mDa, and only the [M]$^+$ and [M+H]$^+$ adducts were considered. If multiple candidates fitted the criteria for the same CNL, they were evaluated based on their likelihood of originating from the fragmentation of compounds within the CNL-*IE* dataset.

### Real data

To evaluate the performance of the CNL model we used a dataset consisting of tea extracts spiked with different combinations of 253 pesticide standards at varying levels of background signal. It should be noted that no analyte from the pesticide standards overlapped with the training sets of the prediction models. This resulted in a total of 66 LC-HRMS chromatograms, including blanks. More details on the analysis can be found elsewhere [37, 38].

The outputs of the suspect screening workflow, which contained both the InChIKeys and the matched fragments of the suspect analytes, were used to evaluate and compare the FP and CNL model. The InChIKeys and a pH value of 2 were used as inputs for the FP model prediction, while the CNL model was used with the measured accurate mass of the feature, the matched fragments and the pH value of 2 as inputs. These predictions enabled us to perform a direct comparison between the outcomes of the two models.

We identified the spiked pesticides in the samples via suspect screening implemented through Universal Library Search Algorithm (ULSA) [51] with a mass tolerance of 10 mDa. The ULSA algorithm uses a suspect list that contains information on both MS1 and MS2 of individual suspect analytes. In total 7114 features were identified using our suspect analysis workflow with at least three match fragments, excluding the parent ion. This approach has been employed for confident identification of suspect analytes in complex samples [52].

### Calculations and code availability

All calculations were performed using a personal computer operating on Windows 10 Pro with a six-core AMD processor and 16 GB RAM. For the calculation of the PaDEL fingerprints, PaDELPy was used [43, 47]. PubChemPy was used for fetching compound metadata and converting molecular identifiers [40]. For the Morgan fingerprints calculation, RDKit was used [45]. CatBoost was used for the model development [48]. For all calculations and plots, Julia v1.8.5 was used. The code for the calculation and selection of the fingerprints, the models and the interpretation of the data is available at: https://github.com/pockos56/IE_prediction-project. The *IE* prediction models were compiled in a Julia package, available at: https://github.com/pockos56/IE_prediction.jl.

## Results and discussion

### Exploration of *IE* dataset

**Fig. 3:**
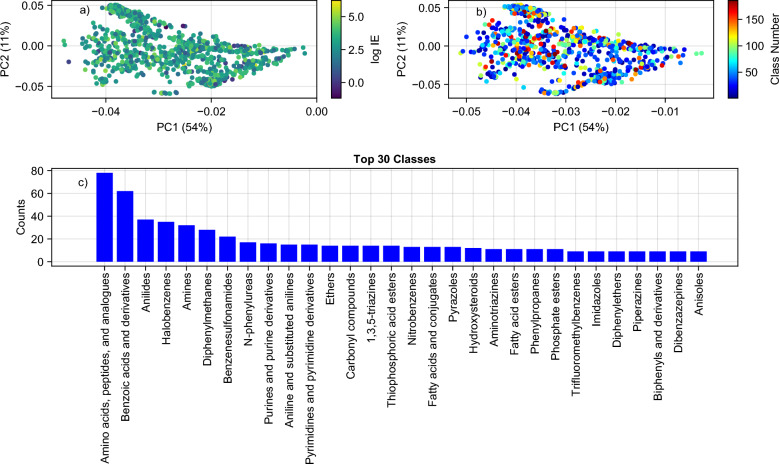
*IE* data summary depicting: **a** the score plots of *IE* dataset for the first two principal components where the colour gradient indicates the experimentally determined log*IE* values, **b** where the colour gradient shows the associated class, and **c** the frequency of occurrence for each chemical class

The *IE* dataset was further explored by performing PCA on the combination of six non-hashed fingerprints as well as the classification based on Classyfire [41] (Figure 3). In total 186 unique chemical classes were present in the *IE* dataset. The most commonly found classes were amino acids/peptide analogues. Overall, the dataset showed a high level of diversity in chemical classes. The score plots showed a random distribution of the *IE* across the PCA space as well as the associated chemical class. This observed trend further indicates the difficulties associated with the *IE* prediction.

### Fingerprint type selection

We established the robust baseline for the CNL model by first training a log*IE* prediction model on molecular fingerprints.

The PubChem fingerprints resulted in the highest $\hbox {Q}^2$ of 0.63, while the other fingerprint types had $\hbox {Q}^2$ values ranging from 0.38 (substructure FP) to 0.61 (MACCS FP). PubChem FPs describe a molecule with a binary bit vector, the bits of which represent the presence of a specific substructure, the minimum count of an element, the neighbours of an atom, etc. [53]. Due to their interpretability, they are easy to trace back to the described molecular feature and to find potential errors in calculation, since their calculation can be easily done by the end user.

**Table 1:** Test set accuracy ($\hbox {Q}^2$) for the optimized FP models

Fingerprint type	$\hbox {Q}^2$
**PubChem fingerprint**	**0.633**
MACCS fingerprint	0.606
CDK extended fingerprint	0.598
2D atom pairs count with compressed PubChem	0.585
PaDEL fingerprints optimized for toxicity prediction	0.564
CDK graph only fingerprint	0.532
Klekota-Roth fingerprint	0.526
2D atom pairs	0.525
Estate fingerprint	0.515
Substructure fingerprint count	0.501
2D atom pairs count	0.497
CDK fingerprint	0.489
Klekota-Roth fingerprint count	0.479
RDKit Morgan fingerprint	0.470
Fingerprint types 1-12	0.460
Substructure fingerprint	0.375

The highlighted FP (PubChem fingerprint) showed the highest $\hbox {Q}^2$ and was therefore selected as the FP type of the final model

### Optimized FP model

For application on identified compounds, an *IE* prediction model was created based on the molecular structure, represented as the PubChem fingerprints. The regression model was optimised for the *IE* dataset and the optimal hyperparameters are shown in Table S1.

**Fig. 4:**
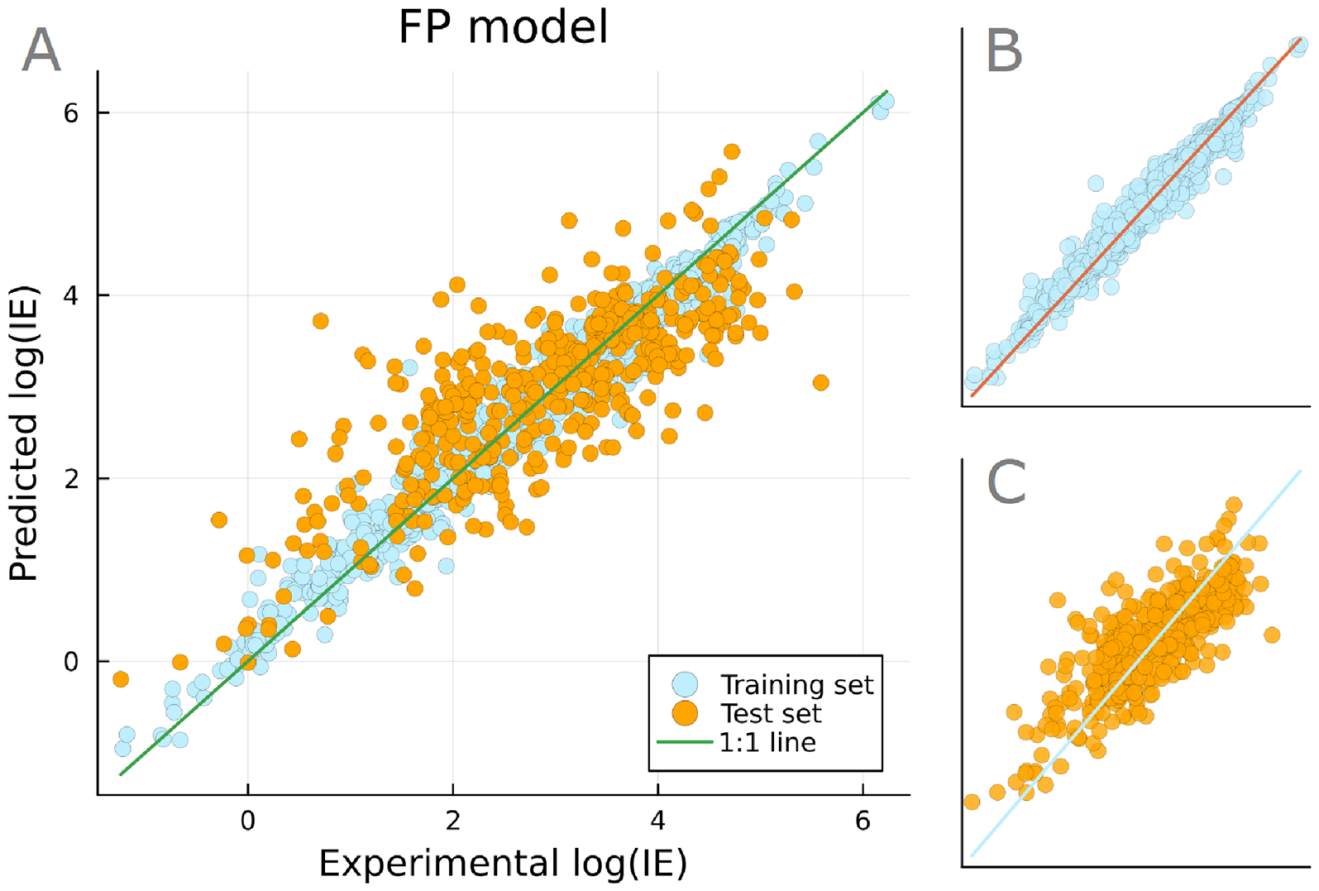
Optimized regression model based on PubChem fingerprints to predict log*IE*. The FP model provides a robust baseline for log*IE* prediction of compounds with a known structure. The main plot (**A**) is a combination of the training subset (**B**) and test subset (**C**) of the model

The model showed a $\hbox {Q}^2$ of 0.63 (Fig. 4) and the mean absolute errors (MAEs) of 0.16 log*IE* units for the training set and 0.55 log*IE* units for the test set. The root-mean-square errors (RMSEs) were also calculated in log*IE* units at 0.22 for the training set and τ0.72 for the test set. Comparing with previous studies, similar accuracy has been achieved with models of similar chemical diversity and conditions. For example, the descriptor-based model by Sepman et al. [27] had RMSE of 0.56 (training set) and 0.81 (test set) log*IE* units. However, models trained on more limited number of data sources and experimental conditions have shown lower errors. For example, the model by Liigand et al. [28] had RMSE of 0.28 (training set) and 0.48 (test set) log*IE* units. Overall, the FP model of this study showed good accuracy for a diverse set of chemicals. Therefore, it should be regarded as the baseline on the expected errors, when the structural information is known.

The compounds with the highest prediction errors were found and examined (Table S2). None of the top 10 outliers were reported in multiple data sources, and therefore their *IE* values were not averaged across different instrumental or measurement parameters. Additionally, most of these compounds show a high extent of charge delocalization, due to multiple resonance structures. Investigating the correlation between the number of possible resonance structures and the absolute prediction error suggested a positive trend, though not decisively (Figure S5). It is suggested that the gained molecular stability is insufficiently predicted by the current model, and therefore the predicted values showed higher errors. This issue should be less pronounced in descriptor-based models, since charge delocalization is a commonly used feature [54]. However, the benefit could be overshadowed by the risk of unreliable descriptors resulting from failed structural optimizations during the descriptor calculation [21–23]. Moreover, no significant correlation was found between the log*IE* prediction errors and the molecular weight of the compounds (Figure S6). Detailed information on the statistical analysis of the resonance structure and molecular weight correlation on the prediction errors can be found in Section S5. Concerning the pH, the residual plots (Figure S3), confirm that the prediction errors are distributed evenly across the entire pH range.

### FP model—important features interpretation

A strong dependence on variables representing the presence of multiple hydrogen and carbon atoms can be observed (>18$\%$ importance), Table 2. They may be interpreted as a measure of the molecular size, which is known to be important for *IE* prediction [54]. Additionally, there are important variables with nitrogen and oxygen atoms ($\ge$21$\%$ importance), which are related to basicity of the molecule, and are considered to have a positive correlation with *IE* [16]. The features containing nitrogen occur more often in top 10 than the ones with oxygen, which can be possibly explained by the higher number of nitrogen-containing compounds in the *IE* dataset. As suggested by previous studies [14], pH has a moderate impact (6.8$\%$ importance) on *IE*.

**Table 2:** The ten most important features and their importance percentage for the optimal FP model

Model feature	Importance (%)
N($\sim$C)($\sim$C)	12.1
$\ge$16 H atoms	11.1
pH	6.8
C-N-C-C-C	5.8
$\ge$8 H atoms	3.3
$\ge$16 C atoms	2.9
C-N	1.9
$\ge$8 C atoms	1.5
$\ge$3 unsat. C rings	1.2
$\ge$1 O atom	1.2

### Optimized CNL model

With an established baseline provided by the FP model, we investigated if the *IE* can be predicted from mass spectral information, expressed as cumulative neutral losses. A prediction model was trained on the CNL-*IE* dataset, which contained CNLs of database mass spectra and reported *IE*s.

**Fig. 5:**
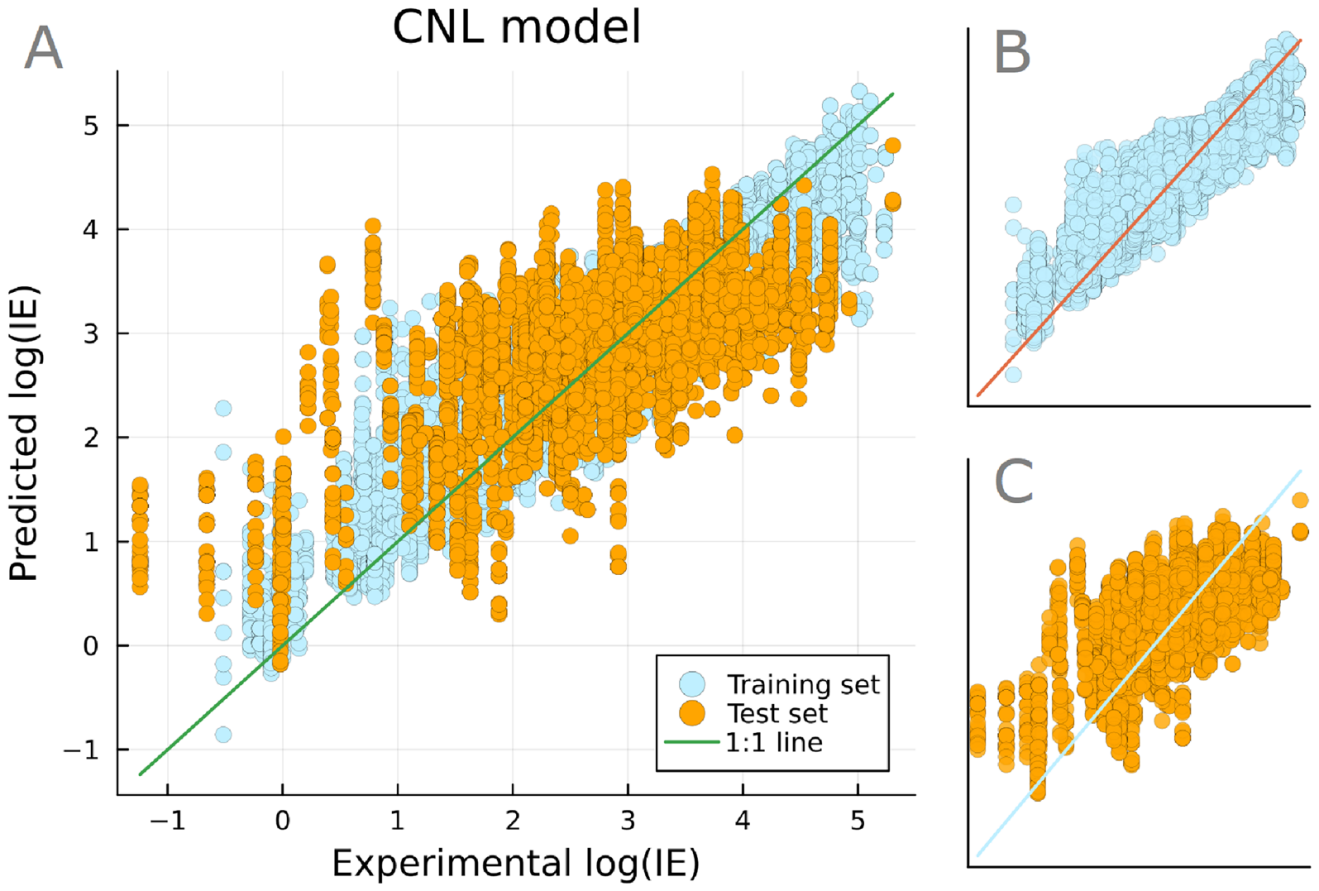
Optimized regression model based on cumulative neutral losses to predict log*IE*. The CNL model provides log*IE* prediction of compounds with an unknown structure. The main plot (**A**) is a combination of the training subset (**B**) and test subset (**C**) of the model

Compared to the FP model, the CNL model optimization also included preprocessing hyperparameters. The model performed the best with at least three fragment ions per spectrum, though no improvement was observed with consensus spectra filtering. A CatBoost regression model was fitted with the optimized hyperparameters (Fig. 5), the detailed information of which is shown in Table S1. The outliers of the CNL and FP models were investigated and compared to assess similar trends. No compound overlapped between the top 10 outliers of the CNL and FP model (Table S2). Nonetheless, both models showed the highest prediction errors in the region of log*IE* below 1, where nine out of the top 10 CNL outliers were found. The CNL outliers had common structural moieties, with four of them containing a carboxyl group and four containing a sulphur atom. The compounds were searched in the LC-MS spectra library of PubChem online database. As of 29-Feb-2024, five compounds had more LC-MS measurements reported in negative ionization mode (ESI-) than in ESI+ and three of the remaining compounds had more than 33$\%$ of the total number of LC-MS spectra measured in ESI-. Thus, the CNL outlier compounds, which have low *IE* in ESI+, have higher *IE* in ESI-, since they are acidic. Additionally, *IE* overestimation is observed for weakly ionized compounds and underestimation for highly ionized ones (Figure S4), similarly to previous studies [27, 28]. While this is partly due to the fact that low log*IE* values are less reproducible, it also suggests the absence of an important underlying parameter from the prediction mechanism and/or high level of variance present in MS2 spectra of a single chemical measured under different instrumental conditions.

Overall, the CNL model had an accuracy lower than the FP model with a $\hbox {Q}^2$ of 0.40 for the test set. The MAE was 0.25 log*IE* units for the training set and 0.62 log*IE* units for the test set, while the RMSE was 0.34 log*IE* units for the training set and 0.79 log*IE* units for the test set. The model’s accuracy is comparable to MS2Quant presented in a research study by Sepman et al. [27]. MS2Quant predicts log*IE* for unidentified analytes from probabilistic molecular fingerprints predicted with SIRIUS+CSI:FingerID from MS1 and MS2 spectra. The log*IE* prediction model showed RMSE of 0.55 (training set) and 0.80 (test set) log*IE* units. The difference of this study and MS2Quant is the use of more eluent descriptors, accounting for the organic modifier and its percentage as well as the buffer type. Nevertheless, the developed models showed similar performance, which could be attributed to error propagation originating from the additional prediction of the molecular fingerprints. In contrast, the current study minimizes error propagation, by direct *IE* prediction based on mass spectral information. Additionally, the reduced number of variables related to the chromatographic conditions used for the model training, enables the use of such models for retrospective analysis of archived data where such information may not be available.

### CNL model—important features interpretation

The mechanism of the CNL model was studied with the feature importance investigation (Table 3). The dominant feature was the monoisotopic mass of the precursor ion, which is related to the molecular size and potentially the surface activity. The second most important feature is the pH with a moderate effect. These two features appeared to be accounted for more than 53% of the feature importance for the *IE* prediction. In other words, the exact mass of the compound and the pH account for most of the variance explained by the CNL model. However, the models are also strongly dependent on the mass spectral variables with a roughly equal (47$\%$) cumulative share of importance. This highlights the necessity to estimate structural information, given the high molecular variability of the training set.

The CNL binning technique complicates finding molecular formulas matching the most important CNL features. Based on possible molecular formulas (Table 3), they are short aliphatic chains (e.g. $\hbox {C}_2\hbox {H}_4$), organic compounds with functional groups (e.g. $\hbox {C}_2\hbox {H}_4$NO, $\hbox {C}_2\hbox {H}_2\hbox {O}_2$), or small inorganic compounds (e.g. $\hbox {CO}_2$, $\hbox {NO}_2$). The latter group consists of the most important features and suggests the presence of a carboxylic acid ($\hbox {CO}_2$) and a nitro moiety ($\hbox {NO}_2$). Aliphatic chains suggest a cleavage at the molecular backbone and could indicate chain length, branches and topological information of functional groups.

**Table 3:** The most important features and their importance percentage for the final CNL model

Model feature	Importance ($\%$)
Monoisotopic mass	39.4
pH	13.9
43.99 ($\hbox {CO}_2$)	2.1
46.01 ($\hbox {NO}_2$)	2.0
28.03 ($\hbox {C}_2\hbox {H}_4$)	1.4
58.01 ($\hbox {C}_2\hbox {H}_4$NO, $\hbox {C}_2\hbox {H}_2\hbox {O}_2$)	1.3

For features representing CNLs of specific m/z values, potential mass fragments are reported. Only features with importance $\ge$ 1$\%$ are displayed

### From known to unknown—explaining the additional prediction uncertainty

The prediction errors of the CNL model were higher than the FP model errors. Nonetheless, a CNL model is independent of compound identification and thus can be applied in cases when no candidate structures are suggested. Similarly to the FP model, given the limited experimental parameters (i.e. pH of the aqueous phase of the eluent) and the inclusion of all different high resolution mass spectrometers and ESI architectures, the CNL model has minimal dependencies. Additionally, since no structural information is required for prediction, the CNL model is suitable to support NTA measurements comprehensively. On these premises, the model accuracy was considered acceptable.

The performance of both the FP and CNL model was evaluated using the conventional approach of comparing predicted values to reference values. Since the FP model is structure-based and contains more information, it provided more accurate predictions. Therefore, it was considered crucial to assess the prediction uncertainty introduced when using the CNL model compared to the FP model, since the FP model has comparable performance to models with similar diversity and can therefore be considered a baseline for predictions provided the molecular structure is known.

**Fig. 6:**
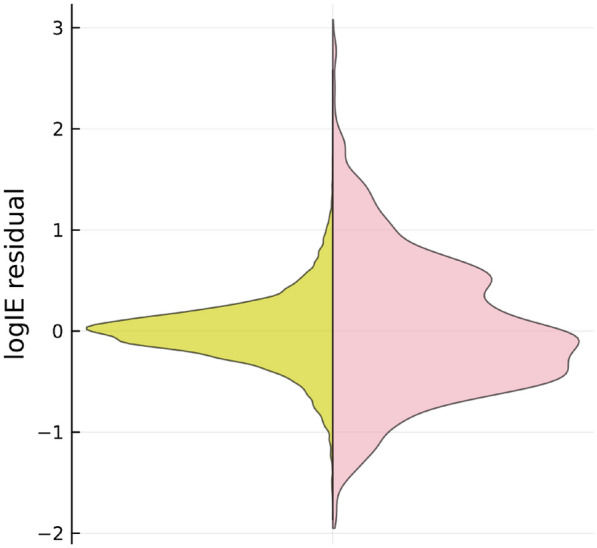
Violin plot showing the residual distribution for the predictions of the CNL model in relation to the predicted values of the FP model. The left (yellow) and right (pink) part of the violin represents the training and test subset, respectively

To quantify the prediction uncertainty introduced from transferring to the CNL model, the accuracy of the CNL model was evaluated based on the predicted values of the FP model. We examined the error distribution of the CNL-based predictions relative to those from the structure-based model (Fig. 6), and the results showed that the RMSE for the test set was 0.43 log*IE* units and 95% of the test set had absolute error of less than 0.91 log*IE* units.

This analysis serves as a reminder that there is a trade-off between information availability and predictive accuracy. Despite the reduced level of information in the CNL model, its performance is reasonable when compared to a model with a higher level of molecular information. The CNL model still provides useful predictions, highlighting its potential for cases where molecular structure is unknown.

### Evaluation with real data

Measurements of tea extracts spiked with a mixture of pesticides at different concentration levels were used for the final evaluation of the CNL model. Specifically, the suspect screening results of 60 LC-HRMS chromatograms were used as benchmark to evaluate the CNL model performance. In total 7114 features with three or more fragments were used for this evaluation.

**Fig. 7:**
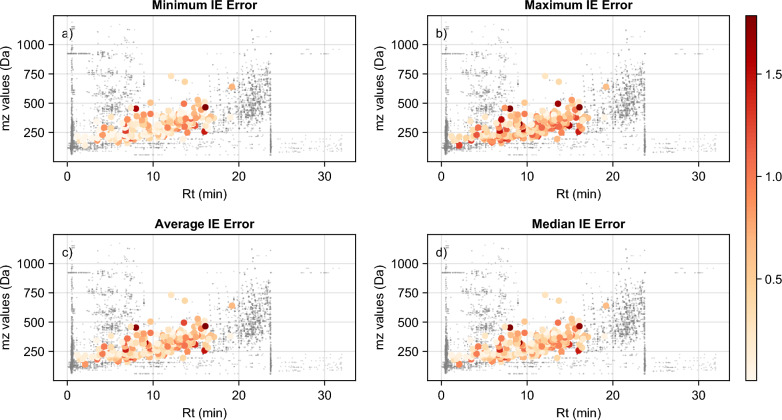
Background chromatogram overlaid by the suspect analyte features used for the evaluation of the models with real data. The panels show: **a** the minimum error, **b** the maximum error, **c** the average error, and **d** the median error for each unique suspect analyte. All data points are shown with a colour scale representing log*IE* units

The average error observed between the FP and CNL model predictions ranged between 0.01 and 1.85 log*IE* units (Fig. 7). This comparison resulted in an RMSE of 0.62 log*IE* units. Overall, the features with higher number of fragments showed higher accuracy. When looking at extreme cases, in most cases they only had three fragments and one or more of those fragments were not included in the original list of selected CNLs, thus not contributing to the model, as these suspect analytes were not part of the CNL or *IE* datasets. This was also observed when looking at the same suspect analyte in multiple samples with different number of fragments and thus a large variance in their prediction bias based on CNL model. The direct comparison of the structure-based model vs CNL model showed that for 3963 out 7114 (56$\%$) cases the error caused by the CNL model was less than a factor of 3 while for 90$\%$ of cases the error was less than a factor of 10 (Figures S7, S8). These results were comparable to the previous studies, even though here no information about the chromatographic gradient and the structural information (i.e. predicted molecular fingerprints) were provided to the model.

## Conclusion

In this study, we successfully created a workflow to predict ionization efficiency for identified and unidentified compounds detected with HRMS. A PubChem fingerprint-based model was developed for the *IE* prediction of compounds with a known structure with the RMSE of the test set at 0.72 log*IE* units. The second part of the workflow employed a CNL-based model to predict log*IE* values for compounds with an unknown structure and showed promising results with the RMSE of the test set at 0.79 log*IE* units. The final evaluation of the model for 7114 suspect analytes, resulted in an RMSE of 0.62 log*IE* units. Overall, these models form a comprehensive tool for *IE* prediction in NTA with or without prior identification of the analyte, and thus make a significant advancement in harnessing the full potential of non-targeted analysis.

The main limitation of the study is the strict applicability to the most widely used ionization mode, ESI+. The ESI- datasets available in literature are substantially smaller. Thus, training a log*IE* prediction model for ESI- or both ESI+ and ESI- measurements was considered inaccessible. In principal, the development of *IE* regression models for ESI- are likely to have promising results with more accurate predictions than the ESI+ models, since ESI- has been found to have less complex source interactions than ESI+ [55], and therefore the *IE* prediction for the former is less challenging.

Despite the elaborate preprocessing and model development, the performance of the CNL model might be improvable. While no additional measurement-related parameters should be provided to the model to maintain the broad applicability of the model, future work might benefit from isolating instrumental categories, such as keeping only the most popular ESI source architecture. Another suggestion would be the inclusion of all formed ion adducts. Although considering all adduct intensities has been reported to improve the prediction accuracy [56], the process involves manual inspection, which prohibits its application in high throughput screening.

Considering the scope of this study, one of the desired properties for the developed models is wide applicability. This requires both molecular and experimental conditions variety in the training datasets to ensure that the models can be applied as universally as possible. While the latter is extensively covered with various experimental parameters, such as mobile phase compositions, instrument manufacturers, ionization source architectures, the former is more limited by the amount of data available in literature. Specifically, the *IE* dataset, an *IE* data collection of thirteen previous studies in a unified scale [27], consists of 1191 unique compounds. Thus, to broaden the applicability domain of *IE* prediction models, it is essential for future research to focus on the analysis and *IE* estimation of more compounds.

Despite these limitations, the current study represents a significant step forward in tackling several challenges encountered in NTA. First and foremost, the ability to predict concentrations in a sample with no prior knowledge of its content could prove to be a useful tool for comprehensive environmental risk assessment. Previous studies have developed toxicity prediction models based on descriptors [22, 57] or mass spectra [38, 58]. Integration of such models in the current or other *IE* prediction workflows could provide a combination of hazard and exposure and a risk estimation of the sample [59, 60], which could lead to the discovery of emerging potentially hazardous substances [61].

Apart from quantification, there are additional applications for the *IE* models. For example, they could contribute to locating more favourable starting points in the process of method development. This can be achieved by predicting *IE*s for a pH range and selecting the pH with the highest *IE*s for a specific set of analytes. Due to the large effect of pH on the *IE*, this process could improve methods by decreasing the limits of detection (LODs) from as early as the method design stage. Ionization efficiency is directly associated with the LOD and therefore the selection of the analytical method for a specific set of (suspected) chemicals could be assisted by LOD prediction [62].

## Supplementary Information

Supplementary material 1. Histogram of the pH distribution in the IE dataset (Figure S1); PCA loadings of the IE dataset exploration (Figure S2); detailed information on the hyperparameter optimization results (Table S1; residual plots of the final fingerprint and CNL model (Figures S3 and S4); list of compounds with the ten highest prediction errors for the FP and CNL model (Table S2); information and plots of the investigation on the correlation of the charge delocalisation and MW with the prediction error of the FP model (Section S5, Figures S5 and S6); and plots on the evaluation of the CNL model performance with real data (Figures S7 and S8).

## Data Availability

The training data was already publicly available. The Julia scripts for the models and plots are publicly available at the GitHub repository here: https://github.com/pockos56/IE The IE prediction models were compiled in a Julia package, available at: https://github.com/pockos56/IE.

## Abbreviations

APC2D: 2D atom pairs count
CDK: Chemistry development kit
CNL: Cumulative neutral loss
ESI: Electrospray ionization
FP: Fingerprint
HRMS: High resolution mass spectrometry
*IE*: Ionization efficiency
InChIKey: International Chemical Identifier
LC: Liquid chromatography
MACCS: Molecular access system
MAE: Mean absolute error
MoNA: MassBank of North America
MW: Molecular weight
NIST: National institute of standards and technology
NTA: Non-targeted analysis
PCA: Principal component analysis
QSPR: Quantitative structure-property relationship
RMSE: Root-mean-square error
ULSA: Universal Library Search Algorithm
