# Supplementary material for: Ionization efficiency prediction of electrospray ionization mass spectrometry analytes based on molecular fingerprints and cumulative neutral losses
Source: J Cheminform. 2025 Dec 6;17:183. doi: 10.1186/s13321-025-01129-7 (PMC12750826; doi:10.1186/s13321-025-01129-7)
Supplement: Supplementary file 1 — Supplementary material 1. Histogram of the pH distribution in the IE dataset (Figure S1); PCA loadings of the IE dataset exploration (Figure S2); detailed information on the hyperparameter optimization results (Table S1; residual plots of the final fingerprint and CNL model (Figures S3 and S4); list of compounds with the ten highest prediction errors for the FP and CNL model (Table S2); information and plots of the investigation on the correlation of the charge delocalisation and MW with the prediction error of the FP model (Section S5, Figures S5 and S6); and plots on the evaluation of the CNL model performance with real data (Figures S7 and S8). [file 13321_2025_1129_MOESM1_ESM.pdf]

Supporting Information for: Ionization Efficiency Prediction of Electrospray Ionization Mass Spectrometry Analytes based on Molecular Fingerprints and Cumulative Neutral Losses

Alexandros Nikolopoulos[1], Denice van Herwerden[1], Viktoriia Turkina[1], Anneli Kruve[2], Melissa Baerenfaenger[3], Saer Samanipour[1,4,5]

[1]Van 't Hoff Institute for Molecular Sciences (HIMS), University of Amsterdam, Amsterdam, 1098XH, The Netherlands.
[2]Department of Materials and Environmental Chemistry, Stockholm University, Stockholm, 11418, Sweden.
[3]Division of BioAnalytical Chemistry AIMMS Amsterdam Institute of Molecular and Life Sciences, Vrije Universiteit Amsterdam, Amsterdam, 1081 HZ, The Netherlands.
[4]UvA Data Science Center, University of Amsterdam, Amsterdam, 1012 WP, The Netherlands.
[5]Queensland Alliance for Environmental Health Sciences (QAEHS), The University of Queensland, Brisbane, QLD 4072, Australia.

Contributing authors: alex.nikolopoulos@student.uva.nl; s.samanipour@uva.nl;

S1 pH distribution - IE dataset

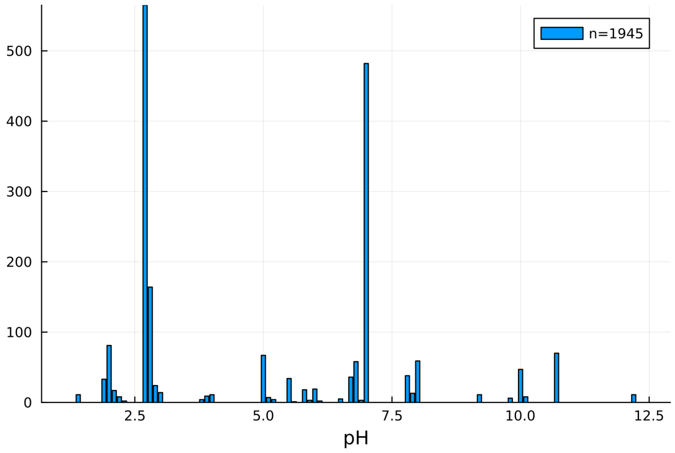

Fig. S1 The pH distribution of the 1945 data points of the IE dataset for the training, validation, and testing of the figure print based model.

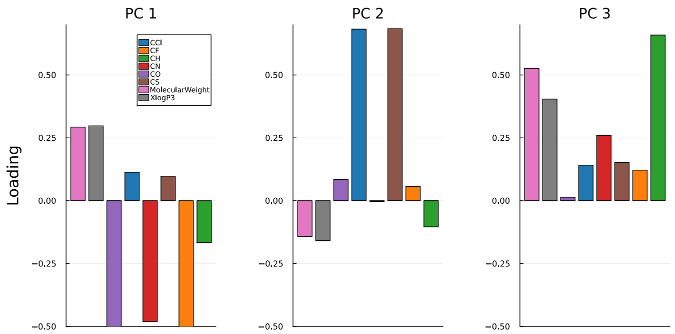

Fig. S2 PCA loadings associated with each variable for the first three principal components.

S2 Hyperparameters of the optimized models

Model	Trees	Learn rate	Leaves	Depth	Subsample	Colsample	Random seed
Fingerprint	700	0.11	4	6	0.58	0.99	3
CNL	1000	0.03	25	7	0.26	0.75	4

Table S1 The hyperparameters of the final FP and CNL models as were used for their training using CatBoost regressors.

S3 Residual plots

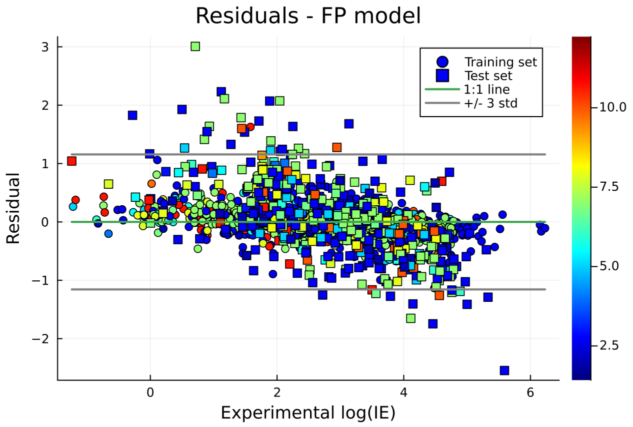

Fig. S3 Residuals of the FP model. The colour of each point represents the pH variable, based on the colour scale on the right side of the plot. The residuals have an even distribution for the whole pH range.

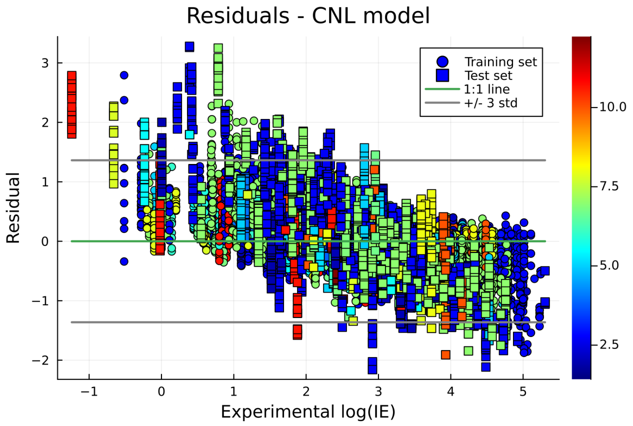

Fig. S4 Residuals of the CNL model. The colour of each point represents the pH variable, based on the colour scale on the right side of the plot. The residuals have an even distribution on the pH range.

S4 Compounds with the highest prediction errors

	FP model	CNL model
1	Tetraethylthiuram disulfide	Ibuprofen
2	Tris(2,4,6-trimethoxyphenyl)phosphine	4-[3-(4-Acetyl-3-hydroxy-2-propylphenoxy)propoxy]phenoxy-acetic acid
3	2,6-dimethoxy pyridine	Dodecanoic acid
4	N-Phenyl-2-naphthylamine	4-methylcatechol
5	5-(3,4-Dichlorophenyl)-8-(methylamino)-5,6,7,8-tetrahydronaphthalene-2-sulfonamide	3-methoxycatechol
6	Norchlorcyclizine	Saccharine
7	3-nitro-1H-1,2,4-triazole	Celecoxib
8	Adipic acid	Isofenphos
9	AM580	Uridine
10	Nikethamide	Methionine

Table S2 The top 10 compounds with the highest prediction errors for the FP and CNL model.

S5 Molecular property correlations with prediction error

The number of possible resonance structures was considered a good estimator of the overall molecular charge delocalisation. Therefore, RDKit v2022.09.3 was used to compute the number of resonance structures of each compound in the test subset of the FP model using their SMILES. The absolute prediction errors for the FP model were also calculated and the Pearson correlation coefficient was calculated at r = 0.13 (Figure S5, blue). The compounds were then grouped by the number of possible resonance structures and their errors were averaged (Figure S5, orange). The Pearson correlation coefficient was calculated at r = 0.50 for the grouped compounds. Consequently, while the analysis on the grouped compounds showed a clear correlation, the analysis on the original data showed no to minimal correlation. Thus, a correlation between charge delocalisation and prediction errors is suggested, but cannot be decisively confirmed.

A similar investigation on the molecular weight was performed. PubChemPy 1.0.4 was used to retrieve the molecular weight of each compound in the test subset of the FP model using their InChIKey. The Pearson correlation coefficient for the molecular weight and the absolute prediction errors was calculated at r = 0.04 (Figure S6,

blue). The compounds were then grouped by their molecular weights and their errors were averaged (Figure S6, orange). The Pearson correlation coefficient was calculated at r = 0.03 for the grouped compounds. The analysis showed clearly that there is no correlation between the molecular weight and the prediction error.

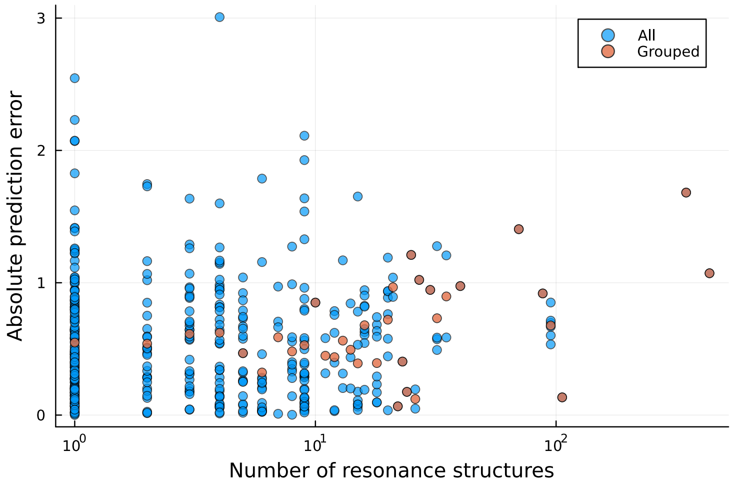

Fig. S5 Scatter plot of the absolute prediction errors (in logIE units) for the test subset compounds of the FP model and the number of possible resonance structures. The blue data points describe all compounds. The orange data points plot describe the average prediction error of the compounds grouped by the number of resonance structures.

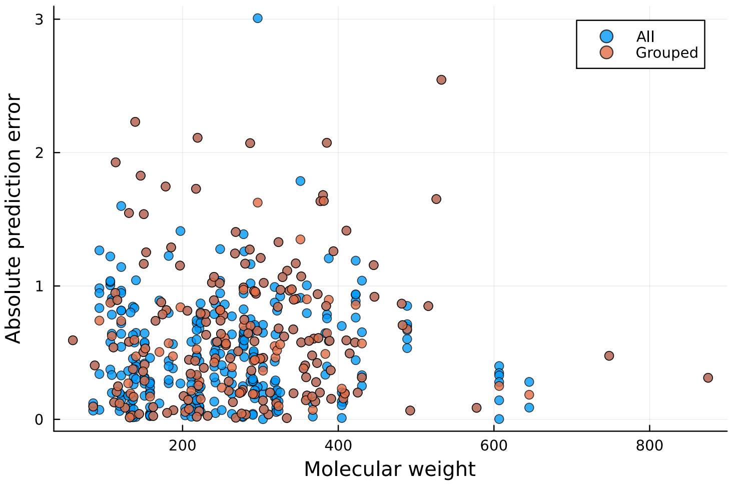

Fig. S6 Scatter plot of the absolute prediction errors (in logIE units) for the test subset compounds of the FP model and their molecular weight. The blue data points describe all compounds. The orange data points plot describe the average prediction error of the compounds grouped by their molecular weight.

S6 Real sample evaluation

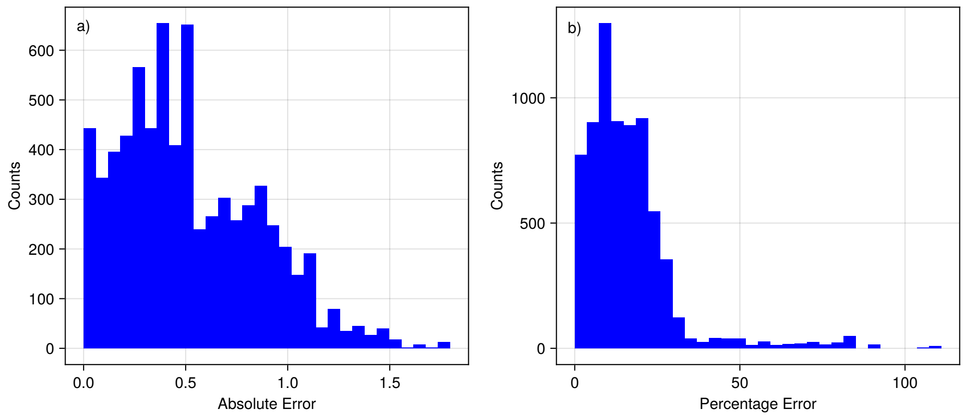

Fig. S7 Distribution of a) absolute error and b) percentage error for the 7114 suspect analytes for IE prediction.

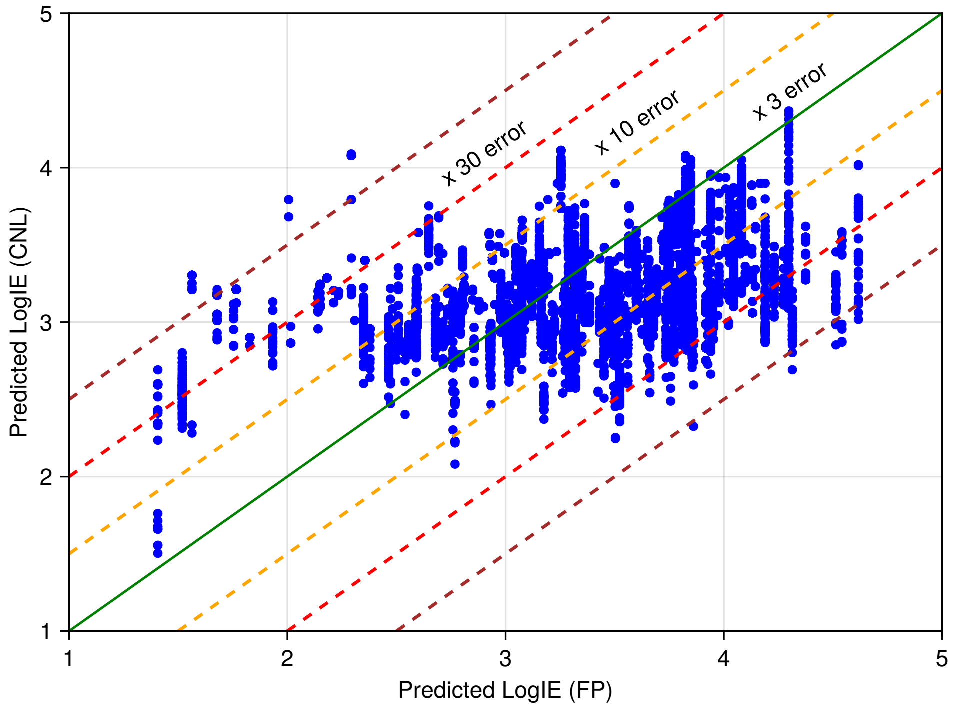

Fig. S8 Scatter plot of predicted log IE of the suspect analytes based on their structure (i.e. FP model) vs based on the fragmentation patterns (i.e. CNL model). Each region represents a bias window when the CNL model is used vs cases where the chemical structure is known.
